# Detection of Immunoglobulin G against E7 of Human Papillomavirus in Non-Small-Cell Lung Cancer

**DOI:** 10.1155/2013/240164

**Published:** 2013-03-03

**Authors:** Raul Storey, Joongho Joh, Amy Kwon, A. Bennett Jenson, Shin-je Ghim, Goetz H. Kloecker

**Affiliations:** ^1^Department of Medicine, University of Louisville, Louisville, KY 40202, USA; ^2^James Graham Brown Cancer Center, University of Louisville, 529 South Jackson Street, Louisville, KY 40202, USA

## Abstract

*Background*. A significant number of non-small-cell lung cancers (NSCLC) have human papillomavirus (HPV) DNA integrated in their genome. This study sought to further establish HPV's possible etiologic link to NSCLC by evaluating an immune response to HPV's oncogene, E7, in patients with NSCLC. *Patients and Methods*. Antibodies (IgG) in serum against E7 for HPV 16 and 18 in 100 patients with NSCLC were examined by enzyme-linked immunosorbent assay (ELISA). *Results*. Sixteen NSCLC patients were found to have a high titration of IgG for HPV oncogenic E7 protein. 23.5% of adenocarcinomas (AC,) and 15.4% of squamous cell carcinomas (SCC) were positive for IgG against HPV E7. HPV-18 (11%) had a slightly higher frequency than HPV-16 (6%). Of the six positive cases for HPV-16, 3 were AC, 2 SCC, and 1 NOS (not otherwise specified). For the 11 HPV-18 positives, 7 were AC, and 4 SCC. The one case with IgG against HPV 16 and 18 was AC. One case had high cross-reactive levels against E7 of HPV 16 and 18. Two (28%) of 7 patients who reported never smoking were positive for HPV, and 12 (13.6%) of 88 smokers were HPV positive. *Conclusions*. The study detected high levels of IgG against E7 in 16% of NSCLC patients. This adds evidence to a potential role of HPV in the pathogenesis of NSCLC.

## 1. Introduction

Lung cancer remains the leading cause of cancer-related deaths worldwide, causing over a million deaths per year worldwide, with an estimated 221,130 new cases and 156,940 deaths in 2011 in the United States [1]. Ten to 20 percent of patients with NSCLC have never smoked [2]. Besides active smoking other etiologic risk factors for NSCLC are known (e.g., toxins, radon, and air pollution); human papilloma virus (HPV) also has been proposed as a potential cause for NSCLC [3]. In his meta-analysis of studies that have looked for detection of HPV in lung cancer, it was noted that HPV detection rates in bronchial carcinomas were found to be highly variable, ranging from 0 percent to 100 percent. On average, of 2,468 bronchial carcinomas, HPV DNA has been reported in 536 (21.7%) of squamous and nonsquamous NSCLC histologies [3]. 

The genome of HPV is a circular double-stranded DNA molecule of approximately 8000 bp. The open reading frames (ORF) include E1, E2, E4, E5, E6, and E7, which are expressed at different stages during epithelial differentiation. The high-risk HPV E6 and E7 ORF encode the viral oncoproteins that are invariably expressed in HPV-positive human cancers. E6 and E7 joint expression is necessary and sufficient for the immortalization of primary human keratinocytes in vitro [3]. Previous studies showed equal expression of E6 and E7 in all cases [4]. E6 and E7 function mainly through decreasing the activities of the retinoblastoma tumor suppression gene and p53 pathway [5]. Low-risk HPV E6 and E7 exhibit much weaker activity than the high-risk HPV E6 and E7 proteins [4]. Levels of these Igs against HPV E7 correlate with the clinical course of cervical cancer [6]. 

E6 and E7 oncoprotein activity also explains the difference in potential carcinogenesis after infection with high-risk HPV versus low-risk HPV [4]. Muller et al. showed an association with seroreactivity to HPV-16 E6-E7 peptides in patients with invasive cervical cancer. Antibodies to HPV-16 E6 and E7 proteins appear to be virus-specific and disease-state-specific markers of HPV-associated cervical cancer [7]. Similar results were found by Rosales et al. [8] Detection of immunoglobulin (IgG) reactivity with the E7 oncoproteins of HPV reflects the effectiveness of the immune response against cervical cancers and their precursor lesions [9]. The reactivity of antibody with the virus oncoprotein appears to be conformationally dependent and specific for HPV type [4, 9]. 

In cervical cancer patients that Ghim and Jenson have followed with this serological test, a predominant IgG response against E7 was associated with periods of tumor stability (dormancy), especially when the cancer was confined to the lymph nodes. An IgG response was usually associated with various rates of progression of tumors, particularly in extranodal sites [6]. 

A recent study using PCR confirmed the presence of 16.7 percent HPV DNA integrated in the DNA of NSCLC [3]. Considering HPV's established causative role in upper airway malignancies and the detection of HPV DNA in a significant number of lung cancers, the question of HPVs etiologic effect on this cancer deserves further study. Since E6/E7 is essential to HPV's carcinogenic effect, E6/7 is expected to be present in non-small-cell lung cancer with a pathogenesis due to HPV. This study examines the presence of antibodies (IgG) in serum against E7 for HPV. Establishing an immune response to HPV in NSCLC would add further evidence to support HPV's etiological role for NSCLC. 

## 2. Material and Methods

### 2.1. Serum Samples

The study utilized 100 unidentified serum samples of NSCLC patients and controls obtained from the Cancer Database and Specimen Repository (CDSR) at the James Graham Brown Cancer Center. Sera from HPV-16 and -18 positive cervical cancer patients were collected as the controls at 4 and 6 different consecutive phases of clinical progression, respectively. However, no detailed clinical histories were available. The sera were used to measure the titration of total IgG against E7 proteins of HPV-16 and -18 by ELISA using recombinant E7 proteins as antigen. 

### 2.2. Antigens

Previously, we successfully generated E7 fusion proteins of HPV16 and 18 using molecular biological techniques. They were fused to either maltose binding protein (MBP) fusion or histidine. E7 genes of HPV16 or 18 were cloned into prokaryotic expression vectors, pMalC2 (New England BioLabs) or pQE30 vector (Qiagen) by PCR cloning technology. The E7 proteins were expressed in *E. coli* and purified as recommended by the manufacturers (NEB and Qiagen, resp.). The expression and quality of these recombinant proteins were examined by SDS-PAGE, immunoblot, and ELISA using standardized molecular biological techniques. The concentration of produced proteins was determined by protein titration using Bio-Rad Protein assay (BioRad). 

### 2.3. Immunological Analysis by ELISA

Direct ELISA method was used to measure IgG titration against E7 of HPV16 and 18. Briefly, approximately 100 ng protein/well in 50 *μ*L bicarbonate buffer (pH 9.6) (Biofluid, Gaithersburg, MD) was coated onto ELISA microplates (Dynatech,VA) for 1 hr at 37°C. After three washings with 200 *μ*L of PBS, 200 mL PBS containing 5% nonfat skimmed milk (5% PBS-SM) was added for 1 hr at 37°C. Then sera diluted to 1/100 in PBS containing 1% bovine serum albumin (1% PBSA) was added for 1 hr at RT followed by the alkaline-phosphatase-(AP-) conjugated goat anti-IgG (H&L chains) of human at a 1/1000 dilution in 1% PBSA for an incubation of 1 hr at 37°C. The reaction was revealed with AP-chromogenic substrate (Sigma-104 p-nitrophenyl phosphate; Sigma, St Louis, MO). The adsorption was measured at 405 nm. Purified MBP proteins were used as control negative. Positive control human sera, which had been previously tested as positive for these HPV oncoproteins, were employed for each experiment. 

### 2.4. Data Analysis

According to the relationship of anti-E7 IgG levels of HPV16 and HPV18, the cut-off points for the positives of HPV16 and/or 18 were selected at the slope changes using the nonlinear least square method. Based on the distribution of ELISA values, the cut-off values of E7 IgG were found at 0.59 (0.045 standard deviation) and 0.48 (0.036 standard deviation) in HPV-16 and -18, respectively. The cut-off points were used to group the patients into the positive and the negative based on whether their immune responses are beyond the cut-off points or not. The samples that showed higher values for both HPV-16 and 18 E7 proteins were selected, and the sample, which showed same distance to both cut-off lines, was decided as the case of double infections of HPV-16 and 18. Statistically significant association of the HPV positive patients with gender, disease stages, smoking status, and age were evaluated by the binary logistic regression method. 

## 3. Results

### 3.1. Demographic Information of Lung Cancer Patients

As shown on Table 1, of the 100 samples, 53% came from males and 47% from females. The patients ranged in age from 42 to 86. Twenty-three percent had a history of cancer other than NSCLC. Additionally, 88% had a history of smoking; 7% never smoked; and in 5% the history of smoking was unknown. Of the samples, 43% were adenocarcinoma (AC), 39% squamous cell carcinoma (SCC), 10% NSCLC not otherwise specified (NOS), 4% large cell carcinoma, and 2% neuroendocrine type (NEC). 

### 3.2. E7 Seropositivity of Control Cervical Cancer Patients

**Figure 1:**
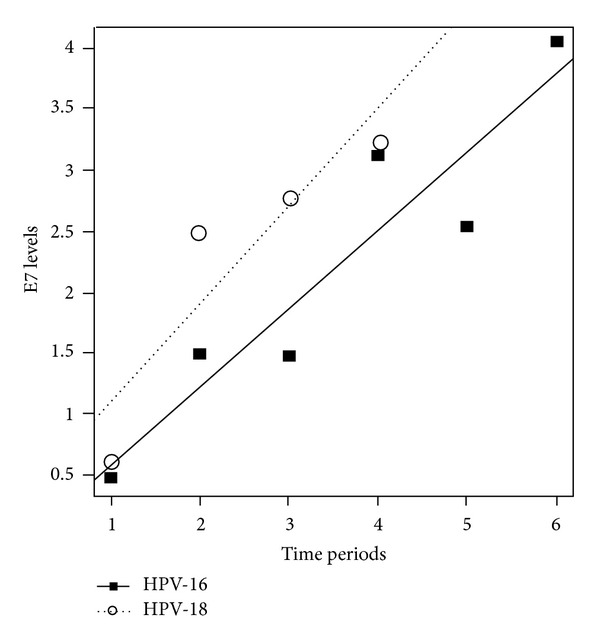
E7 antibody titrations of control HPV-16 and -18 infected patients. The ELISA values were longitudinally obtained from two cervical cancer patients who were infected with HPV-16 and -18, respectively. The solid and dotted lines show the increase of E7 levels over time after the cancers were diagnosed.

The control sera from HPV-16 and -18 positive cervical cancer patients were tested to see if the IgG response to the E7 reflects the prognosis of the cancer. Both sera showed low IgG titrations in the early phase of the illness but showed instant and high immune responses to the oncoprotein in the course of the disease. The titrations increased over time with disease progression except during a short period within the HPV-18 positive group (Figure 1). Because of the unavailability of clinical information of the treatment modalities, the prognosis could not be correlated with the IgG levels, although both patients succumbed to their disease within two to three years. Low titrations appear to be due to the serum collections at early periods of the cancer progression and possibly due to chemotherapeutic treatments that cause immunosuppression. 

### 3.3. E7 Seropositivity of Lung Cancer Patients

**Figure 2:**
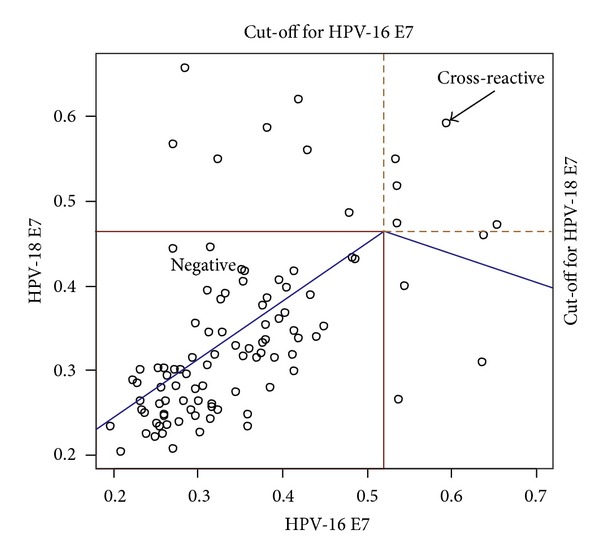
E7 antibody titrations and cut-off points of HPV-16 and/or -18 E7 positive. Scatter plot of ELISA values between HPV-16 and -18 E7s was used to decide positive and negative titrations. The threshold to be the positive was determined at the point where the slope of the predictive line generated by nonlinear optimization was changed. Each cut-off line for HPV-16 and -18 E7 positive was decided, and the patients who have higher titrations than the cut-off were selected as positives. Out of the positives, 5 were located above both cut-off lines, indicating cross-reactivity for both E7s. However, because 4 tended to get close to a cut-off line of one type, only one case (arrow) with the same distance to both cut-off lines was defined as the positive for both.

With the ELISA values of lung cancer patients, the cut-off values of the E7 at 0.59 with 0.045 standard deviations and 0.48 with 0.036 standard deviations in HPV-16 and -18, respectively, were obtained in the statistical distribution (Figure 2). At the given cut-off, 6 and 11 patients were seropositive for the oncogenic E7 proteins of HPV-16 and -18, respectively. Out of the positives, 5 showed the cross-reactions for both E7s, but of those, 4 tended to get close to a cut-off line of one type. Only one case was defined as the positive for both with the same distance to both cut-off lines. Consequently, 16 NSCLC patients were found to have IgG titration for PV oncogenic E7 protein (Table 2).

23.5% and 15.4% of AC and SCC were positive for HPV E7, respectively (Table 2). HPV-18 (11%) had slightly higher frequency than HPV-16 (6%). Of the 6 positive cases for HPV-16, 3 were AC, 2 SCC, and 1 NOS. For the 11 HPV-18 positives, 7 were AC and 4 SCC. The only case with high IgG levels to both types of HPVs, 16 and 18, was AC. Two (28%) of 7 patients who reported never smoking were positive for HPV, and 12 (13.6%) of 88 smokers were HPV positive. 

Twenty-three of the patients had a previous history of at least one other cancer (HPV status at time of recent lung cancer diagnosis in parentheses): uterine (HPV16), rectal, two NSCLC, three bladder (one HPV16), three cervical, laryngeal (HPV16), endometrial (HPV 16) palate, basal cell (HPV16), skin, trigone molar, renal cell (HPV16), prostate (HPV18), tongue (HPV 16/18), melanoma (HPV16), vulva, CLL, and three breast cancers. The seropositivity against E7-IgG was not correlated with other HPV associated cancers.

### 3.4. Statistic Correlation

**Figure 3:**
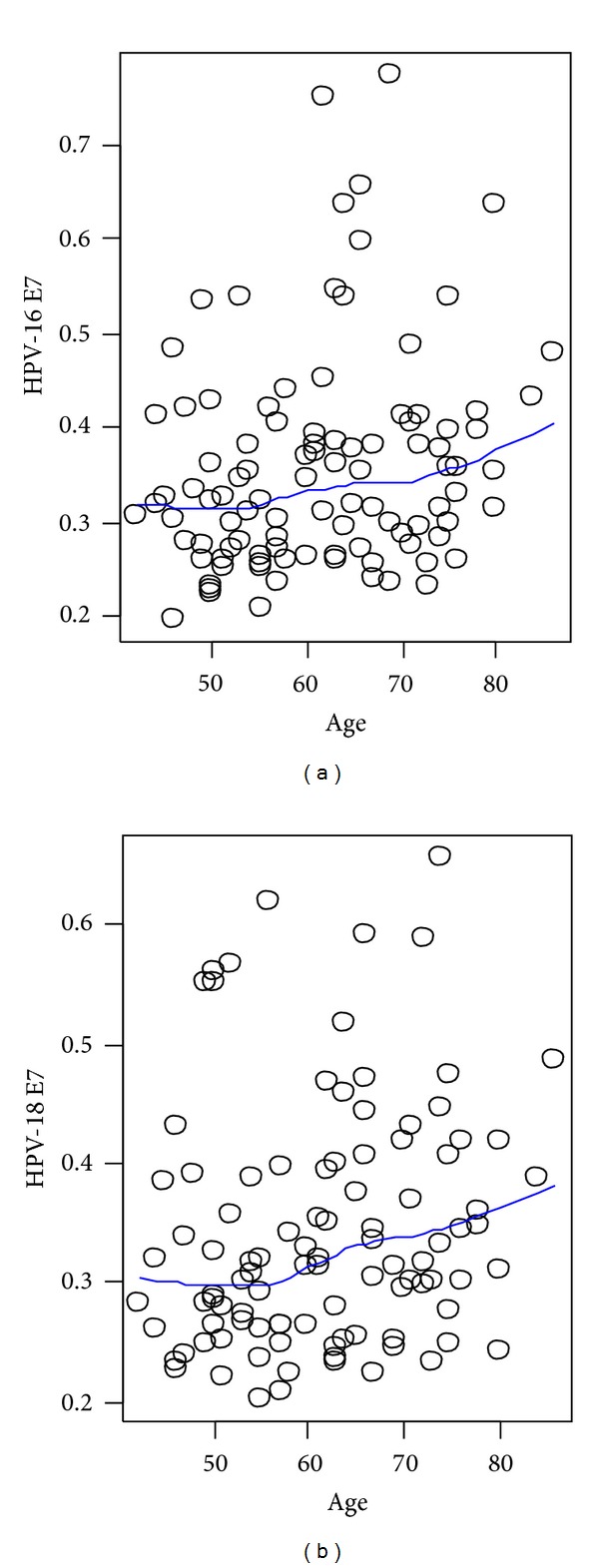
The relationship between E7 titration and age. Scatter plots show distribution of E7 titrations based on ages. The predictive lines based on a nonlinear smoothing method were generated.

Using binary logistic regression any association of HPV infection with gender, disease stages, smoking status, and age of lung cancer patients was examined. The age of the patients showed significant effect on the anti-E7 levels (Figure 3). Both, HPV16 and 18 E7 levels, increased as the patients' ages increased with *P* values of 0.02 and 0.08. However, the other did not show any highly statistically significant association at under *α* = 0.05 with the sample group. 

## 4. Discussion

This study detected IgG against E7 for high risk HPV (type 16 and 18) in 16% of patients with NSCLC. The presence of antibodies against E7 for HPV 16/18 complements previous studies detecting DNA of HPV within NSCLC's genome. The present study found that the age of the patients had a significant effect on the anti-E7 levels. The reason for this is not obvious, but may be due to reinfection or prolonged exposure to the HPV. Never smoking did not correlate clearly statistically with the presence of IgG directed towards HPV-E7, although the number of never smokers was low in this study. IgG against E7 was detected in squamous and nonsquamous histology of NSCLC, which complements the fact that HPV DNA is found in the DNA of both histologies [3, 10]. Patients with a previous history of other cancers, which may be associated with HPV, did not have a higher incidence of IgG levels against E7. It is therefore unlikely that this confounded the results in this study of NSCLC patients.

The presence of HPV DNA alone is not enough to support an active role of the virus in the development of lung cancer, but synthesis of E6 and E7 oncoproteins is a mandatory condition for cell transformation and development of cancer. The early oncoprotein E6 binds and degrades the p53 protein, while the E7 protein binds and inactivates the protein Rb, resulting in abnormal cell proliferation and tumor growth [5, 11, 12]. 

The prevalence of high IgG levels against E7 in lung cancer patients, as well as the fact that the titers increased with age and during the course of the disease, may point to a role of HPV in the pathogenesis of this epidemic disease, that affects also a significant number of never smokers.

In conclusion, the immune response to HPV's oncogene E7 found in NSCLC patients adds to the evidence linking HPV to NSCLC carcinogenesis. In the last years HPV vaccination has been implemented as public policy. The use of these vaccines may also have impact on the incidence of lung cancer. Further studies should be pursued in the future to effects of HPV and vaccines against HPV on NSCLC.

## Figures and Tables

**Table 1:** Summary distribution of 100 lung cancer patients.

Category	Lung cancer patients
Gender	
Male	47 (47%)
Female	53 (53%)
Disease stages	
I	11 (11%)
II	7 (7%)
III	27 (27%)
IV	52 (52%)
Age	61.68 ± 10.6
Current smoker	
Yes	44 (44%)
No	54 (54%)
Unknown	2 (2%)

**Table 2:** Summary of HPV detection with E7 in 100 NSCLC.

		HPV	HPV16	HPV18	Double infection
Non-small-cell lung cancer	100	16% (16/100)	6% (6/100)	11% (11/100)	1% (1/100)
Adenocarcinoma	43	23.5% (10/43)	6.9% (3/43)	16.3% (7/43)	2.3% (1/43)
Squamous cell carcinoma	39	15.4% (6/39)	5.1% (2/39)	10.3% (4/39)	0
NSCLC not otherwise specified	10	10% (1/10)	10% (1/10)	0	0
Large cell carcinoma	4	0	0	0	0
Neuroendocrine type	2	0	0	0	0
